# Novel *MSX1* Gene Variants in Chinese Children with Non-Syndromic Tooth Agenesis: A Clinical and Genetic Analysis

**DOI:** 10.3390/children11121418

**Published:** 2024-11-24

**Authors:** Tingting Ding, Haochen Liu, Guoxia Yu

**Affiliations:** 1 Department of Stomatology, Beijing Children’s Hospital, Capital Medical University, National Center for Children’s Health, Beijing 100045, China; 10989448@bjmu.edu.cn; 2 Department of Prosthodontics, Peking University School and Hospital of Stomatology & National Center for Stomatology & National Clinical Research Center for Oral Diseases & National Engineering Research Center of Oral Biomaterials and Digital Medical Devices & Central Laboratory, Beijing 100081, China; lhc@bjmu.edu.cn

**Keywords:** *MSX1*, non-syndromic tooth agenesis, genotype-phenotype analysis

## Abstract

Background: Tooth agenesis is the most frequently occurring genetic developmental anomaly in clinical dentistry. The *MSX1* gene, essential for tooth development, has been associated with non-syndromic tooth agenesis. This study aims to identify novel *MSX1* variants associated with this condition and to understand their impact on tooth development. Methods: This study involved the genetic analysis of two children presenting with non-syndromic tooth agenesis. Conservation analysis and 3D structural modeling were conducted to assess the pathogenicity of these variants. Additionally, a review of 108 patients with known *MSX1* variants was performed to identify patterns of tooth agenesis. Results: We discovered two novel *MSX1* variants, c.823 T>G and c.890 A>G, located in the second exon of the *MSX1* gene. The identified *MSX1* variants, c.823 T>G and c.890 A>G, were predicted to be pathogenic. Conservation analysis showed that the impacted amino acids are highly conserved across species, and 3D structural analysis indicated potential disruptions to protein function. Among the 108 patients reviewed, a consistent pattern of tooth agenesis was observed, with the most frequently missing teeth being the maxillary second premolars, the mandibular second premolars, and the maxillary first premolars. Conclusions: This research broadens the known range of *MSX1* gene variants and deepens our comprehension of the genetic foundations of non-syndromic tooth agenesis. The findings provide valuable insights for genetic counseling and future research into tooth development, emphasizing the importance of *MSX1* in dental anomalies.

## 1. Introduction

Tooth agenesis is a common congenital developmental anomaly, with incidence rates ranging from 2.2% to 10.1% across various ethnic groups and populations [1,2]. Tooth agenesis can be classified according to the number of missing permanent teeth, excluding the third molars: hypodontia (fewer than six missing permanent teeth), oligodontia (six or more missing permanent teeth), and anodontia (a complete absence of permanent teeth) [3]. Tooth agenesis can manifest as an isolated (non-syndromic) condition or as a component of various syndromes, such as ectodermal dysplasia, Witkop syndrome, or Axenfeld-Rieger syndrome, among others [4,5,6]. Non-syndromic tooth agenesis is more common. Tooth agenesis is a complex multifactorial condition. Tooth agenesis can be caused by genetic factors as well as environmental influences, including trauma, infections, exposure to toxins, and nutritional deficiencies [7,8]. Among these, genetic factors are considered to be the primary causative factors [3,9].

Tooth development is controlled by various signaling pathways, and over 300 genes have been identified as being involved in this process [8]. Currently, variants in several genes have been recognized as contributors to non-syndromic tooth agenesis. These genes include Muscle Segment Homeobox 1 (*MSX1*), Axin Inhibition Protein 2 (*AXIN2*), Paired Box 9 (*PAX9*), Ectodysplasin A (*EDA*), Ectodysplasin Receptor (*EDAR*), EDAR-Associated Death Domain (*EDARDD*), Wingless-Type Mouse Mammary Tumor Virus Integration Site Family Member 10A (*WNT10A*) and 10B (*WNT10B*), Low-Density Lipoprotein Receptor-Related Protein 6 (*LRP6*), and Paired-Like Homeodomain Transcription Factor 2 (*PITX2*), among others [10]. *MSX1* was the first gene discovered to cause non-syndromic tooth agenesis and has remained a focal point of research [11].

The *MSX1* gene belongs to the homeobox gene family and is located at chromosome 4p16.2 [2]. The protein it encodes acts as a transcriptional repressor in the Wnt and BMP4 signaling pathways. *MSX1* plays a crucial role in epithelial-mesenchymal interactions during embryonic development [12]. Mice with *Msx1* gene knockout exhibit symptoms such as halted tooth development, alveolar bone loss, and cleft palate [5]. In humans, variants in the *MSX1* gene are associated with non-syndromic tooth agenesis (OMIM #106600), non-syndromic cleft lip, with or without cleft palate (OMIM #608874), Witkop syndrome (OMIM #189500), and Wolf-Hirschhorn syndrome (OMIM #194190) [13].

The position of tooth agenesis caused by *MSX1* variants is unique. Nieminen’s study showed that the most commonly missing teeth due to *MSX1* variants were the second premolars and the maxillary first premolars [3]. Zheng et al.’s research indicated that the maxillary second premolars, mandibular second premolars, and mandibular first premolars were the three most commonly missing teeth due to *MSX1* variants [1]. Zhao et al.‘s study showed that, in patients with non-syndromic congenital tooth agenesis caused by *MSX1* variants, the most commonly missing teeth were the second premolars and the maxillary first premolars [14].

In this study, we recruited two families with non-syndromic tooth agenesis, where the missing teeth were concentrated in the premolar region. We hypothesized that the congenital tooth agenesis in these patients was caused by *MSX1* gene variants. Therefore, we conducted testing and analysis of gene variants and performed genotype and phenotype analyses.

## 2. Materials and Methods

### 2.1. Subjects Recruitment

The probands from two families affected by non-syndromic tooth agenesis were recruited from the Department of Prosthodontics at Peking University School and Hospital of Stomatology. Dentists from the Department of Pediatric Dentistry at Beijing Children’s Hospital, Capital Medical University, performed oral examinations and took X-rays to confirm the number of missing teeth. Additionally, ophthalmologists, dermatologists, and general surgeons examined the probands to rule out other organ diseases. Oral examinations were also conducted on family members of the patients. Consent was obtained from all participants after informing them about the study. This study was approved by the Ethics Committee of Peking University School and Hospital of Stomatology (PKUSSIRB-202162021). The inclusion and exclusion criteria for the families recruited are shown in Table 1.

### 2.2. Gene Variants Detection

We utilized QIAamp DNA extraction kits (Qiagen, Hilden, Germany) to isolate genomic DNA from the venous blood of the participants. Whole exome sequencing (WES) was conducted on 9 members from 2 families using the Illumina X10 platform by Beijing Angen Gene Medicine Technology (Beijing, China). Initially, genes related to craniofacial development were annotated from the sequencing results [15]. Subsequently, all nonsynonymous single nucleotide variants (SNVs) and insertions/deletions (indels) with a minor allele frequency (MAF) of 0.01 or less were filtered using the following databases: dbSNP (http://www.ncbi.nlm.nih.gov/projects/SNP/snp_summary.cgi, accessed on 1 October 2024) and gnomAD (http://gnomad.broadinstitute.org, accessed on 1 October 2024). The impact of the identified variants on protein function was predicted using the following software tools: SIFT (http://provean.jcvi.org/index.php, accessed on 1 October 2024), PROVEAN (http://provean.jcvi.org/index.php, accessed on 1 October 2024), PolyPhen-2 (http://genetics.bwh.harvard.edu/pph2/, accessed on 1 October 2024), CADD (https://cadd.gs.washington.edu/, accessed on 1 October 2024), and REVEL (https://sites.google.com/site/revelgenomics/, accessed on 1 October 2024).

### 2.3. Sanger Sequencing

To confirm the results of the WES analysis, we utilized polymerase chain reaction (PCR) to amplify the two exons of the *MSX1* gene (NM_002448) in the participants. Primers are available upon request. The variants were validated and their segregation within the families was analyzed. The PCR products were sent to Tsingke Biological Technology (Beijing, China) for Sanger sequencing.

### 2.4. Conservation Analysis

We downloaded the protein sequences of MSX1 from various species (*H. sapiens*, *P. troglodytes*, *M. mulatta*, *C. lupus*, *B. taurus*, *M. musculus*, *R. norvegicus*, *G. gallus*, *D. rerio,* and *X. tropicali*s) from the NCBI database (https://www.ncbi.nlm.nih.gov/, accessed on 1 October 2024) for conservation analysis. Multiple sequence alignment was performed using Molecular Evolutionary Genetics Analysis Version 11 (MEGA 11.0) software to analyze the conservation of the MSX1 protein across species.

### 2.5. Protein 3D Structure Analysis

We used the Alpha Fold Protein Structure Database (https://alphafold.ebi.ac.uk/, accessed on 1 October 2024) to predict the 3D structure of the MSX1 protein. Next, we used PyMol v2.1 software (DeLano Scientific, San Carlos, CA, USA) to analyze the changes in the 3D structure of the protein caused by these two variants (p.Ser275Ala and p.Tyr297Cys) and predict their impact on the function of the MSX1 protein.

### 2.6. Literature Review and Statistical Analysis

To analyze the clinical characteristics of tooth agenesis caused by *MSX1* variants, we reviewed previous studies on the non-syndromic tooth agenesis phenotype in MSX1 variant patients [1,11,13,14,15,16,17,18,19,20,21,22,23,24,25,26,27,28,29,30,31,32,33,34,35,36,37,38,39,40,41,42]. Based on the HGMD and PubMed databases, we summarized data from 105 patients with detailed records of tooth agenesis. Including the three patients from this study, a total of 108 patients were analyzed (Supplement Table S1). Statistical analysis was conducted using GraphPad Prism software (v.10.1). We used the Chi-square test (or Fisher’s exact test) for single-factor analysis to compare the frequencies of missing teeth at different positions. The data are expressed as mean ± standard deviation (SD), with a sample size of n = 3. A *p*-value of less than 0.05 was considered to indicate statistical significance.

## 3. Results

### 3.1. Clinical Phenotype

**Figure 1:**
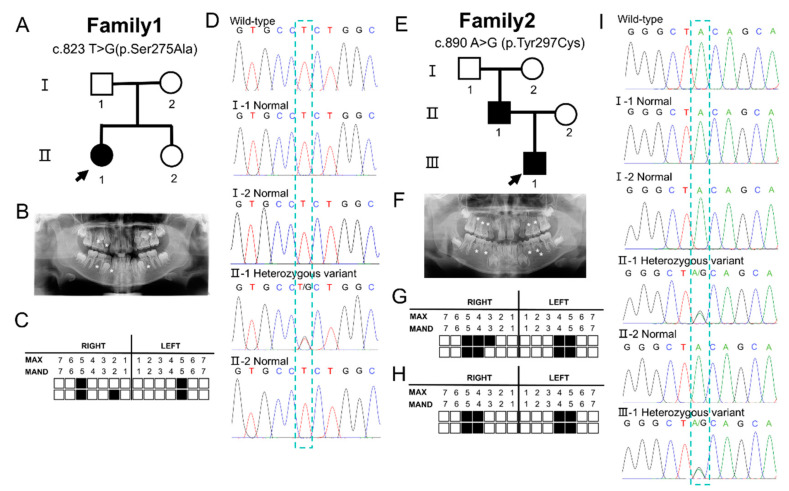
Clinical information and sequencing results from two families. (**A**) Pedigree of Family 1. (**B**) Panoramic tomogram of the proband in Family 1 (II:1). (**C**) Diagram showing the pattern of missing teeth in the proband in Family 1 (II:1). (**D**) The proband in Family 1 (II:1) has the *MSX1* missense variant (c.823 T>G), while the other family members have the wild-type sequence. (**E**) Pedigree of Family 2. (**F**) Panoramic tomogram of the proband from Family 2 (III:1). (**G**) Diagram showing the pattern of missing teeth in the proband in Family 2 (III:1). (**H**) Diagram showing the pattern of missing teeth in the proband’s father in Family 2 (II:1). (**I**) The proband (III:1) in Family 2 and his father r(II:1) carry the MSX1 missense variant (c.890 A>G), while the others have the wild-type sequence. The arrow in the pedigree diagram marks the proband. Asterisks on the panoramic tomograms and the black squares in the diagrams denote congenitally missing permanent teeth. Max, maxillary; Mand, mandibular.

Family 1 is a two-generation family (Figure 1A). The proband #1 is a 9-year-old female with the congenital absence of five permanent teeth (Figure 1B,C), but no other organ abnormalities, and is thus diagnosed with non-syndromic tooth agenesis. The parents and younger sister of the proband are healthy, with no abnormalities detected.

**Figure 2:**
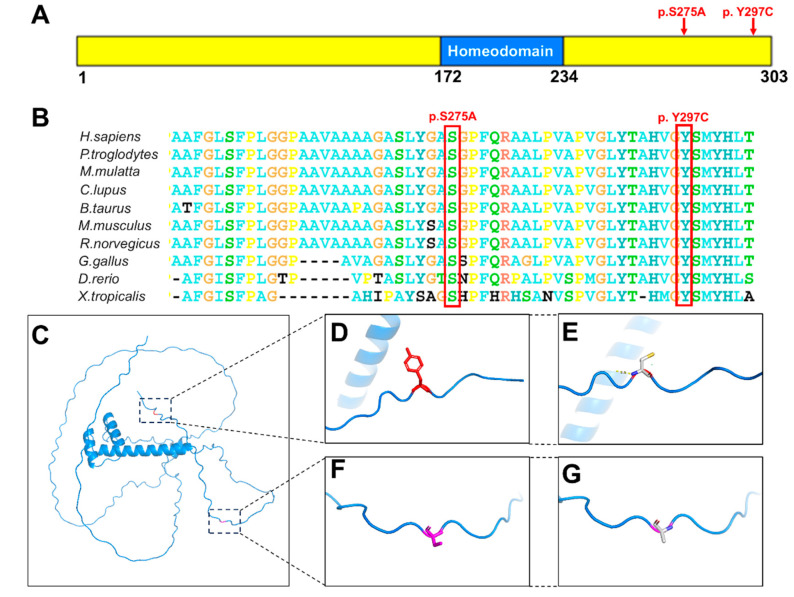
Variant position, conservation, and 3D structure analysis. (**A**) Model of the human MSX1 protein structure and the distribution of the two novel variants (p.Ser275Ala and p.Tyr297Cys) identified in this study. (**B**) Conservation analysis of the changed amino acids (275Ser and 297Tyr) in the MSX1 protein. (**C**) The Alphafold-predicted structure of the human msx1 protein. (**D**) Higher magnifications of 297Tyr. (**E**) Higher magnifications of amino acid substitution (p.Tyr297Cys) in the MSX1 protein. (**F**) Higher magnifications of 275Ser. (**G**) Higher magnifications of amino acid substitution (p.Ser275Ala) in the MSX1 protein.

Family 2 is a three-generation family (Figure 2E). The proband #2 is a 7-year-old male with the congenital absence of nine permanent teeth (Figure 1F,G), but no other organ abnormalities, and is thus diagnosed with non-syndromic tooth agenesis. The proband’s father has the congenital absence of eight permanent teeth (Figure 1H). The proband’s mother, grandfather, and grandmother are healthy, with no abnormalities detected.

### 3.2. Identification of Two MSX1 Variants

Through WES analysis and Sanger sequencing, we identified a novel heterozygous variant in the *MSX1* gene (c.823 T>G; p.Ser275Ala) in the proband of Family 1. Sequencing results for the three unaffected individuals in this family were consistent with the wild type (Figure 1D). These results are consistent with familial co-segregation. Utilizing the results from SIFT, PROVEAN, PolyPhen-2, CADD, and REVEL, and adhering to the 2015 American College of Medical Genetics and Genomics (ACMG) guidelines for pathogenic variant classification, c.823 T>G (p.Ser275Ala) is predicted to be pathogenic (Table 1).

In Family 2, we identified a novel heterozygous missense variant in the *MSX1* gene (c.890 A>G; p.Tyr297Cys) in both the proband and his father, who also has congenital tooth agenesis. Sequencing results for the three unaffected individuals in this family were consistent with the wild type (Figure 1I). These results are consistent with familial co-segregation. Based on the results from SIFT, PROVEAN, PolyPhen-2, CADD, and REVEL, and following the 2015 ACMG guidelines for pathogenic variant classification, c.890 A>G (p.Tyr297Cys) is classified as pathogenic (Table 2).

### 3.3. Conservational and Structural Analysis

The two variants identified in this study, c.823 T>G (p.Ser275Ala) and c.890 A>G (p.Tyr297Cys), are located in the second exon of the *MSX1* gene, at the C-terminal end of the MSX1 protein (Figure 2A). Conservational analysis indicates that the amino acids at positions Ser275 and Tyr297 are highly conserved across different species, suggesting that variations at these sites may significantly impact the protein’s function.

Three-dimensional structural analysis reveals that the residue at position 297 is a tyrosine, which has an aromatic side chain (Figure 2D). This side chain can stack with other amino acids, contributing to the stability of the protein structure. The variant residue is cysteine, whose side chain can form a disulfide bond with another cysteine (Figure 2E), providing robust structural support for the protein. At position 275, the residue is serine, which has a neutral side chain (Figure 2F). The variant residue is alanine, which has an aliphatic side chain (Figure 2G) and is hydrophobic. This change from a neutral to a hydrophobic side chain could alter the local environment and affect the protein’s function.

### 3.4. Tooth Agenesis Phenotype Associated with MSX1 Variants

**Figure 3:**
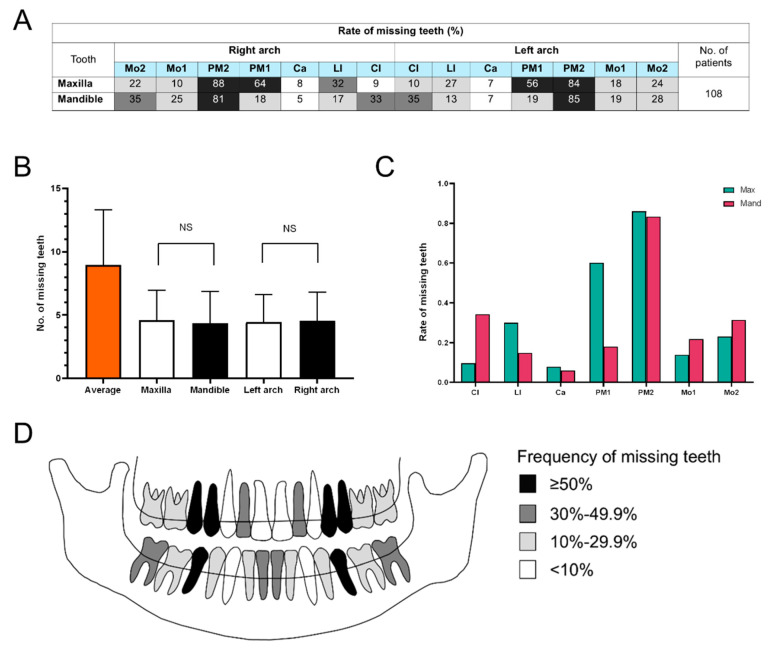
Characteristics of non-syndromic tooth agenesis related to *MSX1* variants. (**A**) Frequency of tooth agenesis at each position. (**B**) The number of missing teeth per dental arch (mean ± SD). (**C**) Frequency of tooth agenesis at seven positions in the maxillary and mandibular dentitions, respectively. (**D**) Pattern diagram showing the frequency of tooth agenesis at each position, with darker colors indicating a higher probability of agenesis. NS: No Significance; Mo2: second molar; Mo1: first molar; PM2: second premolar; PM1: first premolar; Ca: canine; LI: lateral incisor; CI: central incisor; Max: maxillary; Man: mandibular.

We compiled data from a total of 108 patients with non-syndromic tooth agenesis caused by *MSX1* variants. The frequency of tooth agenesis at each dental position was calculated (Figure 3A). On average, patients with non-syndromic tooth agenesis associated with *MSX1* had an average of 8.96 missing teeth. No significant differences were observed in the number of missing teeth between the maxilla and mandible (*p* > 0.05), or between the left and right dental arches (*p* > 0.05) (Figure 3B). We then combined the data from the left and right dental arches and calculated the frequency of tooth agenesis at each dental position in both the maxilla and the mandible. We found that the teeth most frequently missing were the maxillary second premolars (86%), the mandibular second premolars (83%), and the maxillary first premolars (60%) (Figure 3B). The teeth least frequently missing were the mandibular canines (6%), the maxillary canines (8%), and the maxillary central incisors (10%) (Figure 3C). Based on the frequency of tooth agenesis, we constructed a pattern diagram related to the *MSX1* gene variant and non-syndromic tooth agenesis. The darker the color, the more likely the tooth at that position was to be missing (Figure 3D).

## 4. Discussion

The congenital absence of teeth caused by *MSX1* variants is usually inherited in an autosomal dominant manner [1,11,21]. In this study, the proband in Family 1 is a heterozygous missense variant (c.823 T>G; p.Ser275Ala). The genotype and phenotype of the proband’s parents and sister are normal. It is inferred that the variant of the proband is a de novo variant, which aligns with the autosomal dominant inheritance pattern. In Family 2, the proband and the proband’s father both have the phenotype of non-syndromic tooth agenesis and the genotype is a heterozygous missense variant (single nucleotide variant) (c.890 A>G; p.Tyr297Cys). The genotypes of all the other people in the family, who are healthy, are wild-type, inferring that the variant of the proband’s father is a de novo variant, which is then inherited by the proband, which is indicative of the autosomal dominant inheritance pattern. Two main types of *MSX1* gene variants are associated with non-syndromic tooth agenesis: missense variants and frameshift variants [1,14]. The research of Zheng et al. shows that the missense variants of *MSX1* all occur in the highly conserved area of the second exon [1]. The two newly discovered variants from this study are located in the second exon of the *MSX1* gene and the encoded amino acids are highly conserved during evolution. The results of this study are consistent with this phenomenon. The highly conserved area of amino acids is a structurally or functionally important area and is a hotspot for discovering missense variants [43]. The results of this study further confirm that the highly conserved area of the second exon of *MSX1* is a hotspot for missense variants.

MSX1 protein is composed of multiple structural domains, among which the most important is the homeodomain, which is a highly conserved DNA-binding structural domain [44]. Variants occurring in the homeodomain region can affect the nuclear localization of the MSX1 protein, thereby affecting cell function and having a large impact on protein function [26]. The two variants in this study, p.Ser275Ala and p.Tyr297Cys, are not located in the homeodomain region. However, the 3D structural analysis shows that the 275th amino acid of the MSX1 protein changes from polar serine to hydrophobic alanine, which may disrupt the formation of hydrogen bonds and post-translational modifications and affect the stability, folding of the protein, and the interaction with other proteins or DNA, thereby changing its regulatory function in the development process. The tyrosine at the 297th position of the MSX1 protein is replaced by cysteine, which may introduce the possibility of disulfide bond formation, which has a significant impact on the stability of the protein, DNA-binding ability, and interaction with other proteins. These changes may disrupt the normal function of MSX1 as a transcription factor, leading to developmental abnormalities and diseases. Therefore, we infer that the two variants, p.Ser275Ala and p.Tyr297Cys, found in this study have an impact on protein function, which is lighter than the variants in the homeodomain region. The clinical condition of the patients is non-syndromic tooth agenesis, and the number of missing teeth is less than or equal to the average. Of course, the specific pathogenic mechanism is still unclear and needs to be further studied in the future.

Variants in multiple genes can cause tooth agenesis. Tooth agenesis caused by different gene variants not only differs in the number and location of missing teeth, but may also be accompanied by other systemic symptoms [3,8,45]. Summarizing the phenotypic characteristics of tooth agenesis caused by each gene variant will provide great help for clinical treatment and genetic counseling. This study compiles cases of non-syndromic tooth agenesis attributed to *MSX1* variants reported in previous research. We found that the teeth most likely to be missing in non-syndromic tooth agenesis caused by *MSX1* variants are the maxillary second premolars, the mandibular second premolars, and the maxillary first premolars; the least likely missing teeth are the mandibular canines, the maxillary canines, and the maxillary central incisors. This result aligns with the conclusions of previous research [1,14]. This study has drawn a pattern diagram of congenital tooth agenesis caused by *MSX1* (Figure 3D), which helps readers understand this phenotype more intuitively.

Multiple gene variations can lead to non-syndromic tooth agenesis. In our previous study, we found that dual variants in the *EDA* and *WNT10A* genes can exacerbate the phenotype of congenital tooth agenesis [46]. Additionally, our research has shown that biallelic variations in the *WNT10A* gene can worsen the tooth agenesis phenotype [47]. Therefore, we hypothesize that variations in multiple genes can intensify the tooth agenesis phenotype, but the specific mechanisms require further investigation. Currently, there are no reported cases involving multiple gene variations, including *MSX1* and other genes.

In the process of tooth development, *PAX9* and *MSX1* co-regulate downstream target genes through mutual interaction [48]. *PAX9* can directly or indirectly affect the expression of *MSX1*, thereby affecting tooth morphogenesis; *MSX1* may regulate the function of *PAX9* by affecting cell proliferation and differentiation [49]. This regulation is crucial for the correct formation of teeth [50]. However, the phenotypes of non-syndromic tooth agenesis caused by variants in *PAX9* and *MSX1* are different. The research of Liu et al. shows that the teeth most likely to be missing in tooth agenesis caused by variants in *PAX9* are the maxillary second molars, the mandibular second molars, and the maxillary first molars [51]. This is significantly different from the tooth agenesis pattern of the *MSX1* gene variant. This shows that the regulatory mechanisms of teeth at different positions are different. This reminds us that each tooth has its own unique and complex development mechanism.

This study has several limitations. We did not conduct functional experiments, so we cannot elucidate the specific mechanisms by which *MSX1* gene variants lead to congenital tooth agenesis. Based on previous research results, we are currently constructing conditional *MSX1* knockout mice to further investigate the role of the *MSX1* gene in tooth development. Additionally, due to the unique characteristics of human tooth development, we also plan to create *MSX1* knockout dogs and miniature pigs for more in-depth mechanistic studies.

## 5. Conclusions

In general, this study is the first to report two new variants of the *MSX1* gene (c.823 T>G and c.890 A>G), further expanding the variant spectrum of this gene. We further summarized the tooth agenesis pattern caused by *MSX1* variants in non-syndromic tooth agenesis and drew a pattern diagram, which helps us further understand the etiology of tooth agenesis and provide help for clinical diagnosis and genetic counseling.

## Figures and Tables

**Table 1:** Inclusion and exclusion criteria for the families recruited.

Inclusion Criteria	Exclusion Criteria
Congenital absence of permanent teeth (excluding third molars)	Other organ diseases present besides congenital tooth agenesis
Detailed dental records confirming congenital tooth agenesis	No detailed dental records confirming congenital tooth agenesis
Signed informed consent for study participation	Declined to participate in the study

**Table 2:** Predicted functional impact of the two *MSX1* variants.

Subjects	Exon	Nucleotide Change	Protein Change	Variation Type	SIFT	PROVEAN	PolyPhen-2	CADD	REVEL	ACMG Classification
Proband#1	2	c.823 T>G	p.Ser275Ala	Missense	Damaging	Neutral	probably damaging	Damaging	Disease variants	Pathogenic
Proband#2	2	c. 890 a>G	p.Tyr297Cys	Missense	Damaging	Deleterious	probably damaging	Damaging	Disease variants	Pathogenic

## Data Availability

The original contributions presented in this study are included in the Supplementary Materials; further inquiries can be directed to the corresponding authors.

## Supplementary Materials

The following supporting information can be downloaded at: https://www.mdpi.com/article/10.3390/children11121418/s1.
